# Validation of Remote Dielectric Sensing (ReDS) in Monitoring Adult Patients Affected by COVID-19 Pneumonia

**DOI:** 10.3390/diagnostics11061003

**Published:** 2021-05-31

**Authors:** Federico Mei, Alessandro Di Marco Berardino, Martina Bonifazi, Lara Letizia Latini, Lina Zuccatosta, Stefano Gasparini

**Affiliations:** 1 Respiratory Diseases Unit, Azienda Ospedaliero-Universitaria “Ospedali Riuniti”, 60126 Ancona, Italy; fedusmei@gmail.com (F.M.); Alessandro.DiMarcoBerardino@ospedaliriuniti.marche.it (A.D.M.B.); lety.94@hotmail.it (L.L.L.); lina.zuccatosta@ospedaliriuniti.marche.it (L.Z.); s.gasparini@univpm.it (S.G.); 2 Department of Biomedical Sciences and Public Health, Università Politecnica delle Marche, 60126 Ancona, Italy

**Keywords:** COVID-19 pneumonia, lung edema, fluid monitoring, computed tomography, remote dielectric sensing

## Abstract

Remote dielectric sensing (ReDS) is a non-invasive electromagnetic wave technology which provides an accurate reading of lung fluid content, and it has been reported as a valid tool in monitoring heart failure patients. Considering that morphological alterations in COVID-19 include pulmonary edema, the purpose of the present study was to evaluate the reliability of ReDS technology in assessing the excess of lung fluid status in COVID-19 pneumonia, as compared to CT scans. In this pilot single center study, confirmed COVID-19 patients were enrolled on admission to an intermediate care unit. Measurements with the ReDS system and CT scans were performed on admission and at weeks 1 and 2. Eleven patients were recruited. The average change in ReDS was −3.1 ± 1.7 after one week (*p* = 0.001) and −4.6 ± 2.9 after two weeks (*p* = 0.006). A similar trend was seen in total CT score (−3.3 ± 2.1, *p* = 0.001). The level of agreement between ReDS and CT changes yielded a perfect result. Statistically significant changes were observed in lactate dehydrogenase, lymphocytes, and c-reactive protein over 2 weeks. This pilot study shows that ReDS can track changes in lung involvement according to the severity of COVID-19. Further studies to detect early clinical deterioration are needed.

## 1. Introduction

In December of 2019, an outbreak of a novel coronavirus disease (COVID-19) occurred in Wuhan, a city in the Chinese province of Hubei, and, thereafter, it dramatically spread worldwide [1,2,3,4,5,6]. COVID-19 can present with a wide spectrum of clinical manifestations, ranging from mild or no symptoms (80%) to severe pneumonia with acute respiratory distress syndrome (ARDS) (20%) [2]. Pathological features include diffuse alveolar damage, proteinaceous exudate, focal reactive hyperplasia of pneumocytes with patchy inflammatory cellular infiltration, and progressive extracellular lung water collection, leading to pulmonary edema. Vascular changes like hyperplasia/dilatation of alveolar capillaries, new angiogenesis, endothelialitis, and thrombotic microangiopathy are also described [7,8,9,10,11,12].

Chest computed tomography (CT) is crucial to assess the pattern as well as extent of lung lesions, and the most common radiological pictures include pure “ground-glass” opacities (GGO), GGO with reticular and/or interlobular septal thickening, GGO with consolidations, crazy paving, and pure consolidations [13,14,15]. A bilateral lung involvement with a peripheral subpleural distribution is present in the majority of cases [13]. Different CT features are also associated with patient prognosis, with mixed patterns being more common in severe cases with poor outcomes [13,16]. The thoracic ultrasound technique has been also shown to play a complementary key role in the diagnosis and follow-up of COVID-19 pneumonia [17,18], since it allows to easily identify subpleural alterations and to monitor them over time [19,20]. Moreover, it offers the advantage of being low cost, non-ionizing, and available at bedside [21,22]. However, TUS does not provide information on the whole lung parenchyma, as it allows to visualize only subpleural, peripheral involvement.

A further non-invasive device, intended to measure the dielectric properties of tissues, mainly determined by fluid content, is electromagnetic energy-based technology, named remote dielectric sensing (ReDS™, Sensible Medical Innovations Ltd., Netanya, Israel). In detail, ReDS™ is an FDA- and CE-cleared device that measures lung fluid content quickly, in absolute terms, providing objective and reproducible indices of volume status [23,24,25,26]. It was first tested in patients with heart failure [23], showing a strong correlation with computed tomography (CT)-measured lung water [25], invasively determined pulmonary artery wedge pressure [27,28], and clinical evolution. ReDS has also been demonstrated to be helpful in guiding the management of patients [24,26].

Considering that morphological alterations in COVID-19 include pulmonary edema, the purpose of the current pilot study was to evaluate the reliability of ReDS technology in assessing the excess of lung fluid status in COVID-19 pneumonia, as compared to CT scans, and to explore its potential role in monitoring the clinical evolution of these patients by measuring the correlations with longitudinal changes of radiological features and selected serological parameters.

## 2. Materials and Methods

### 2.1. ReDS Technology

**Figure 1:**
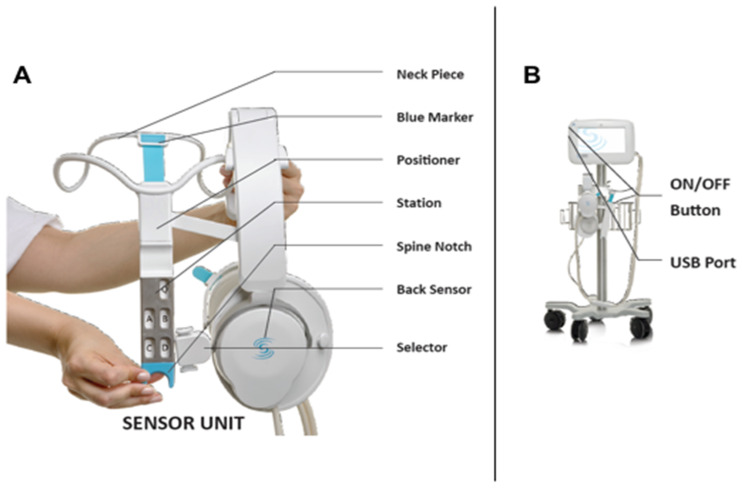
(**A**) ReDS wearable vest; (**B**) Monitor console.

**Figure 2:**
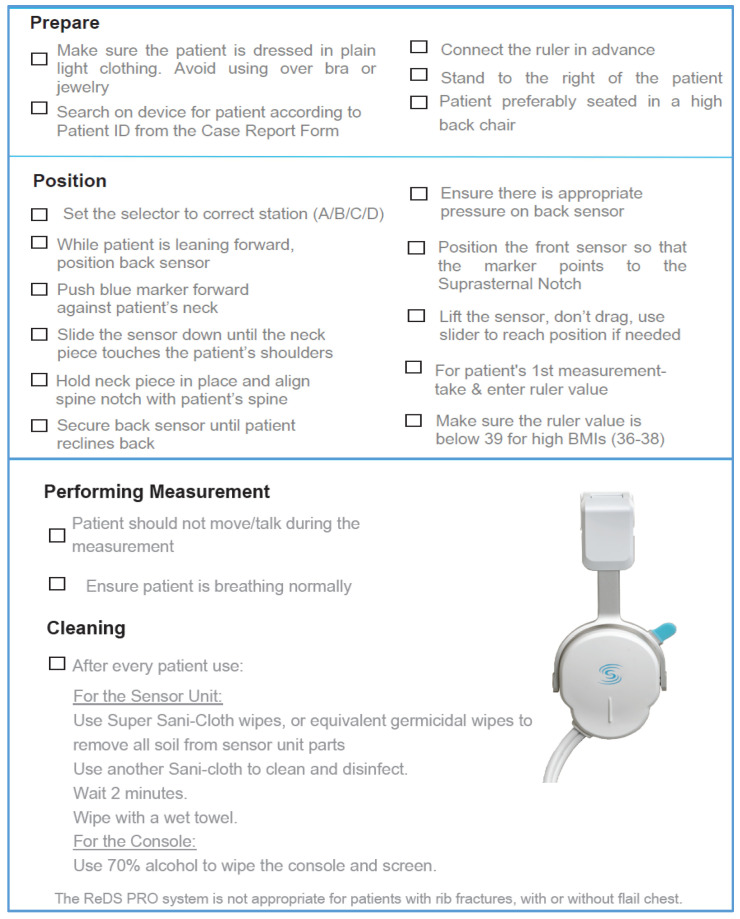
Operating instructions of the ReDS™ Pro System device.

The ReDS™ Pro System is a point of care device, consisting of two sensors in a clip configuration, that is applied on the patient for 45 s long measurement. When applying the device, the sensors are positioned on the front and back of the patient’s thorax with no need for direct skin contact, allowing measurements to be performed through light clothing (Figure 1A). The device is connected via a cable to a bedside monitor console (Figure 1B). A comprehensive checklist of operating instructions is provided in Figure 2.

Low-power electromagnetic signals are transmitted through the thorax between the sensors, and the intercepted signals reflect the dielectric properties of different tissues. Since lung tissue is primarily composed of water and air, with water having a very high dielectric coefficient and air having the lowest dielectric coefficient, the average dielectric coefficient reflects the percentage of lung tissue fluid content. The normal value of lung fluid volume is between 20% and 35%. The full range of lung fluid volume reported by the ReDS system spans from 15% to 60%.

The ReDS technology has FDA and CE approvals, and it is indicated for assessment of fluid overload. Currently, it is being used for the measurement and monitoring of lung fluid content in heart failure patients [7,8,9,10,11,13].

### 2.2. Patients and Clinical Information

The study protocol was approved by the local ethical committee (Comitato Etico Regione Marche, n. 2020131, 7 April 2020) and performed at the University Hospital “Ospedali Riuniti”, Ancona, Italy.

Consecutive patients, aged ≥ 18 years, admitted to the sub-intensive care unit between 10 April (first day of availability of ReDS device) and 4 May 2020 (day of admission of last patient during the first wave of the COVID-19 pandemic in Italy) with a diagnosis of pneumonia due to severe acute respiratory syndrome coronavirus 2 (SARS-CoV-2), confirmed by reverse transcription polymerase chain reaction (RT-PCR) performed on a naso-pharyngeal swab or bronchoalveolar lavage, were prospectively enrolled in the present study according to eligibility criteria. Exclusion criteria were thorax deformity, recent (last 3 months) chest trauma or rib fractures, presence of a pacemaker, inability to remain seated during measurements, and inability to give an informed consent.

Demographic factors and selected clinical characteristics were collected for all cases. These included: age, sex, smoking history, height, weight, comorbidities, gas exchange values (pO_2_/FiO_2_)P/F), white blood cells count (WBC), lymphocytes count (Ly), d-dimer, interleukin 6 (IL-6), C-reactive protein (CRP), lactate dehydrogenase (LDH), B-type natriuretic peptide (BNP), and need for oxygen support, including non-invasive mechanical ventilation (nIMV). nIMV was indicated with SpO_2_ < 92% on oxygen therapy 15 L/min Fi O_2_ 50%, while mechanical ventilation (IMV) was indicated when respiratory rate was above 25/min and/or signs of acute respiratory failure despite nIMV. The follow-up period was the time from admission to the date of discharge or death. Main clinical outcomes (death, discharge) were collected during the follow-up period.

All patients were measured with the ReDS system three times during their in-hospital stay: on admission (t0), at 7 days (t1), and at 15 days (t2) later. Each measurement was performed by trained operators (F.M. and A.D.M.B.) according to standard procedure.

### 2.3. Radiological Assessment

All patients underwent chest CT scans, combining high resolution (HRCT) and pulmonary angiogram protocol (CTPA), on admission and 2 weeks later. Each CT scan was evaluated independently and by two pulmonologists with 10 and 12 years of experience in order to define the main radiological pattern and total extent of parenchymal involvement. Final decisions were reached by consensus. Each CT evaluation by reporting pulmonologists was blind to the ReDS data.

The prevalent morphological patterns at the CT scan were classified as follows: 0 = pure GGO; 1 = crazy paving; 2 = GGO with consolidations; 3 = consolidations; 4 = consolidations with interseptal thickening.

The extent of parenchymal involvement at CT was scored by visually evaluating the percentage of lesions’ involvement at the lobar basis according to a 5-point categorical scale, using the following scoring system: 0 = none (0%), 1 = minimal (1–25%), 2 = mild (26–50%), 3 = moderate (51–75%), 4 = severe (76–100%). The total severity score (TSS) was reached by summing the five lobe scores (range from 0 to 20); severe parenchymal lung involvement was defined as a TSS value of 8 or more [29].

### 2.4. Statistical Analysis

The primary endpoint was the concordance between CT scores and ReDS values. The secondary endpoint was the concordance between ReDS and laboratory results. The parameters’ values and their differences were compared using descriptive statistics, *t*-test, and correlation methods. Statistical significance was declared when the *p*-value was found to be less than or equal to 0.05 (two sided). All statistical analyses were performed using the SAS statistical software or equivalent statistical software.

## 3. Results

A total of eleven patients were included in the present study. Demographic and clinical characteristics at baseline (i.e., in hospital admission) for all patients are summarized in Table 1. The median age of the study cohort was 63 ± 11 years, the majority of patients were male (72.7%) and had never smoked (54.5%), and the median level of BMI was 25.5 ± 3.7 kg/m^2^. Comorbidities were present in more than two thirds of patients (72.7%) and blood hypertension was the most prevalent condition, whereas none reported a pre-existing cardiac disease. The mean value of P/F was 152.8 ± 70.1. All patients received oxygen support and three of them required nIMV (27.7%). D-dimer, LDH, and CRP were altered in most of the study cohort (599.3 ± 873.4 mg/dL, 321.45 ± 103.6 U/L, 4.7 ± 4.3 mg/dL mean values, respectively), while BNP was normal for all patients (average value: 72.5 ± 38.9 ng/mL).

Chest CT scans revealed diffuse pulmonary alterations in all patients and most of them (81.8%) presented with severe involvement (TSS ≥ 8), with a mean TSS at baseline of 10.1. Consolidations only (36.4%) and ground glass opacities plus consolidations (36.4%) were the most prevalent morphological pattern, followed by crazy paving and pure GGO (respectively, 18.2% and 9.1%). No further abnormalities were observed, except for a mild unilateral pleural effusion in one patient.

The mean value of ReDS measurements at baseline was 29.6 ± 7.6%, without significant difference between left and right lung. ReDS measurements greater than the upper limit of normal (i.e., ≥35%) in at least one lung were present in 5 patients (45%).

**Figure 3:**
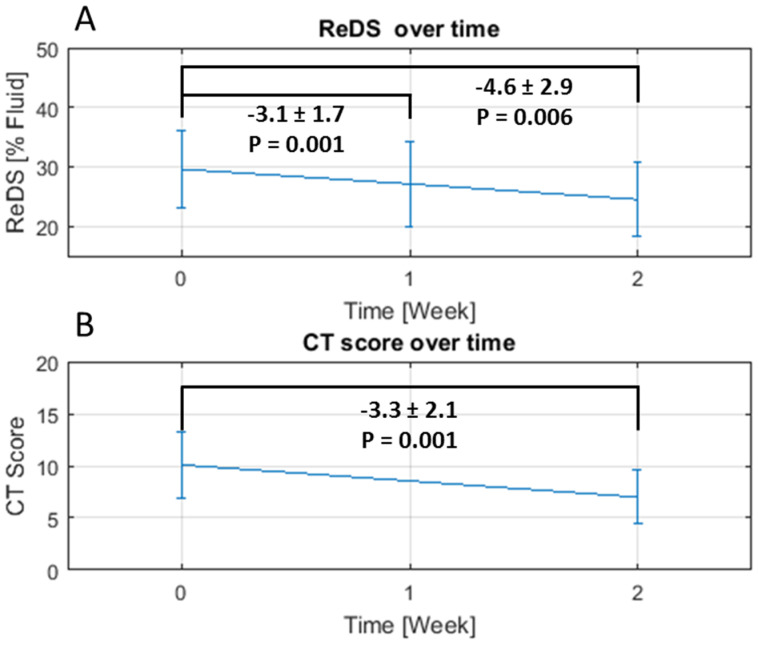
Average change in ReDS measurements (**A**) and CT score (**B**) over time.

**Figure 4:**
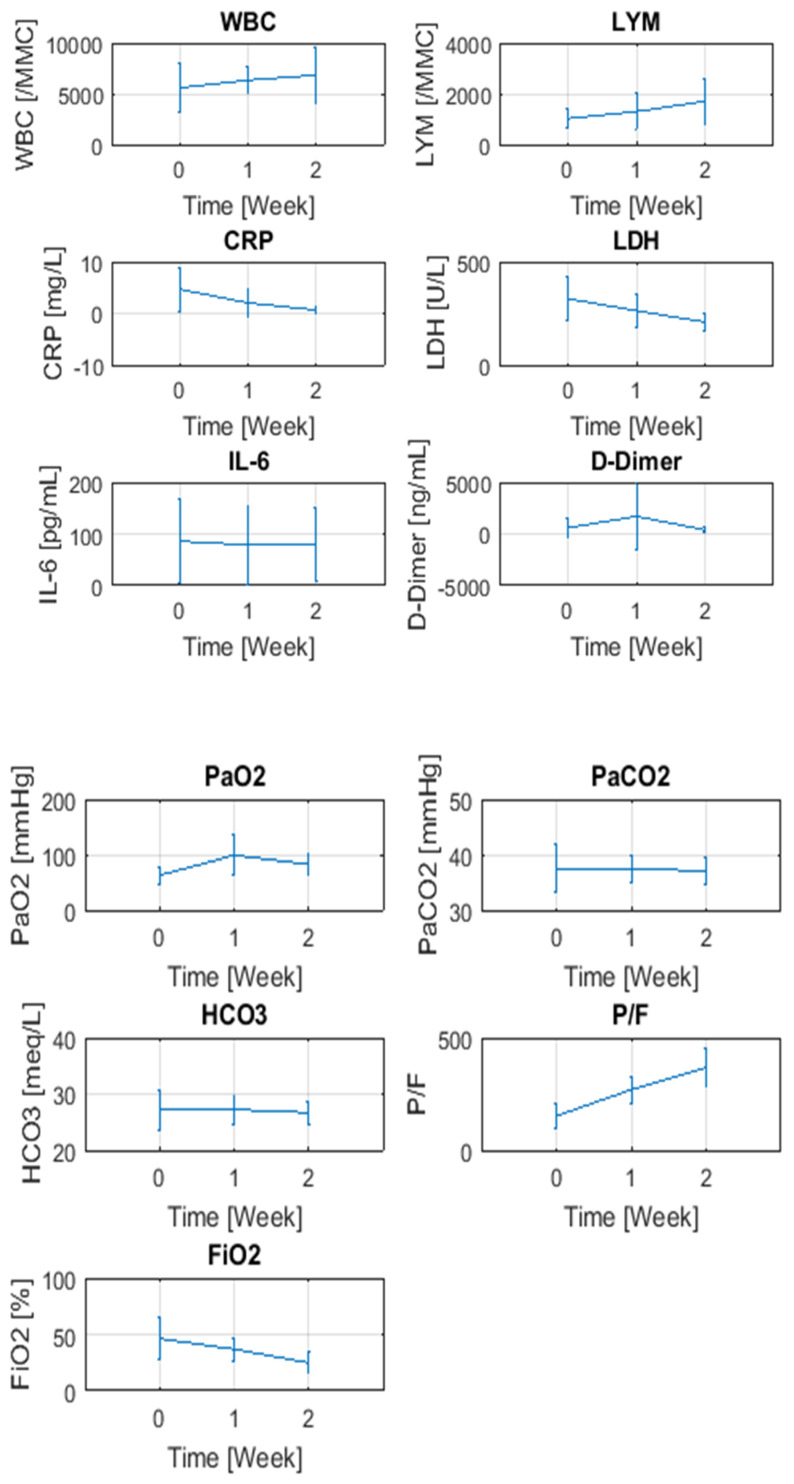
Changes in blood tests and clinical indicators over time.

All patients had repeated ReDS measurements and blood tests at days 7 and 15 and a CT scan at day 15. The average change in ReDS measurements after one week was −3.1 ± 1.7 (% lung fluid, *p* = 0.001) and 4.6 ± 2.9 (% lung fluid, *p* = 0.006) after two weeks (Figure 3A). A similar trend was seen in the total CT score (−3.3 ± 2.1, *p* = 0.001) (Figure 3B). The level of agreement between ReDS and CT 15-day trends yielded a perfect result (100%). Statistically significant changes were observed in LDH, lymphocytes, and CRP over 15 days. Changes in the partial pressure of oxygen (PaO_2_), fraction of inspired oxygen (FiO_2_) and their ratio (P/F) were also significant (Figure 4, Table 2). All the patients were discharged after clinical improvement.

## 4. Discussion

The present study firstly describes a novel approach to assess and monitor lung fluid excess in patients hospitalized for COVID-19 pneumonia using ReDS technology. Our findings overall show a not negligible proportion of patients with fluid overload in at least one lung, and a significant longitudinal change of ReDS readings over time, which were strongly correlated with CT score and selected clinical features. Notably, ReDS measurements corresponded to clinical evolution throughout hospitalization, supporting the potential utility of ReDS in monitoring COVID-19 pneumonia.

Pulmonary fluid overload during COVID-19 pneumonia is thought to be the expression of lung edema due to hyper-inflammation, one of the leading pathological mechanisms causing respiratory failure during the first “exudative phase” [7]. This is mainly characterized by desquamation of alveolar epithelial cells, alveolar-capillary barrier injury with red blood cell extravasation, and intense inflammatory cells infiltration in the intra-alveolar space and around small vessels. The subsequent proliferative phase is dominated by fibroblast and myofibroblast proliferation, leading to acute fibrinous organizing pneumonia and parenchymal remodeling. It is worth underlining that these stages often occur simultaneously [7,8].

The excellent correlation between ReDS readings and CT-measured lung water was already established in previous studies. In particular, Amir et al. demonstrated a high correlation between the ReDS system and CT results in patients with heart failure [25] and they successively evaluated the role of longitudinal readings in order to reduce the re-admission rate, providing encouraging results [24]. Lala et al. were also able to show a reduction of re-admission rate by utilizing ReDS to manage heart failure patients during post-discharge follow-up visits [30], and Bensimhon et al. showed that patients with high ReDS reading at discharge are at a higher risk of being readmitted. Moreover, ReDS measurements have been shown to rule out elevated pulmonary artery wedge pressure (PAWP), as lung fluid content correlates well with PAWP with a high negative predictive value [27].

A major advantage of the ReDS™ system is that it is safe, non-invasive, non-ionizing, easy to use, and requires minimal patient collaboration. Moreover, it is available at the bedside, leading to reducing the contagious risk for health-workers during patient transportation and the need to sanitize larger areas of equipment (just the probe instead of the whole radiological suite). Italy was the first Western country to experience this unexpected and devastating pandemic, with a multitude of infected patients admitted to emergency departments over few weeks. This led to an urgent and profound re-organization of the national healthcare system, including the widespread adoption of telemedicine and remote counselling [31,32], as well as the creation of dedicated wards and alternative pathways for in-hospital patient transport. In this context, tools able to quantify and monitor the evolution of pneumonia at bedside, avoiding repeated CT scans for longitudinal evaluations, are, indeed, extremely helpful. Compared to TUS, it offers the advantage of providing information on fluid excess across the whole parenchyma and not only in the subpleural areas.

Major strengths of this pilot study include the novelty of results, the prospective nature of the study design that determined an accurate collection of data for each patient, and the objective measurements of the main outcome.

The study has some limitations. First, the clinical study was strictly observational, by study design, and therefore the role of the ReDS™ system in improving outcomes could not be determined. Second, the choice of a cutoff point may be criticized due to a lack of prospective validation. We chose to use this cutoff according to prior published works suggesting that normal lung water content is 20–35%. However, the main purpose of this pilot study was not to determine the proportion of COVID-19 patients with significant fluid excess, but to assess role of this device as a monitoring tool, evaluating how ReDS values (regardless of being above or below cut-off value) correlated to CT features and to clinical evolution. A third limitation is the limited number of patients included.

## 5. Conclusions

To our knowledge, this is the first pilot study that aimed to measure lung fluid overload in COVID-19 pneumonia with ReDS technology, suggest that ReDS technology has potential for monitoring these patients at bedside and avoiding repeated CT scans for longitudinal evaluations.

Larger, multicenter studies are needed to confirm these findings, to determine the appropriate timing for longitudinal evaluation, and to explore the clinical impact of the ReDS™ system on the management of patients hospitalized with COVID-19 pneumonia.

## Figures and Tables

**Table 1:** Distribution of demographic and clinical characteristics at baseline (on hospital admission).

	Total
Variables	*n* = 11
Gender, *n* (%)	
Males	8 (72.7)
Females	3 (27.3)
Age, years (median ± SD)	63 ± 11
Smoke, *n* (%)	
Never	6 (54.5)
Current/Former	5 (45.5)
BMI (median ± SD)	25.5 ± 3.7
Comorbidities, *n* (%)	
Yes	8 (72.7)
No	3 (27.3)
ReDS (% fluid ± SD)	29.6 ± 7.6
P/F (mean ± SD)	152.8 ± 70.1
HRCT TSS, *n* (%)	
mild (total score < 8)	2 (8.2)
severe (total score ≥ 8)	9 (81.8)
HRCT pattern, *n* (%)	
pure GGO	1 (9.1)
crazy paving	2 (18.2)
GGO with consolidations	4 (36.4)
consolidations	4 (36.4)
consolidations + interseptal thickening	0
WBC, per mmc (mean ± SD)	5589.1 ± 2343.3
Lym, per mmc (mean ± SD)	1027.3 ± 396.4
D-dimer, mg/mL (mean ± SD)	599.3 ± 873.4
LDH, U/L (mean ± SD)	321.45 ± 103.6
CRP, mg/dL (mean ± SD)	4.7 ± 4.3
BNP, ng/mL (mean ± SD)	72.5 ± 38.9
Oxygen therapy, *n* (%)	
No	0
Yes	11 (100)
nIMV, *n* (%)	
No	8 (72.7)
Yes	3 (27.3)

Footnotes. BMI: body mass index; BNP: B-type natriuretic peptide; CRP: reactive C protein; GGO: ground glass opacity; HRCT: high resolution computed tomography; LDH: lactate dehydrogenase; LYM: lymphocytes count; nIMV: non-invasive mechanical ventilation; P/F: pO_2_/FiO_2_; WBC: white blood cells count.

**Table 2:** Average change in selected clinical and serological parameters over time.

Parameter	15-Day Change (Δ)	*p*-Value
ΔWBC	959 ± 3333.6	0.39
ΔLYM	705.1 ± 597.5	0.005
ΔCRP	−4.4 ± 4.2	0.009
ΔLDH	−106.4 ± 94.6	0.006
ΔIL-6	−9.7 ± 75.1	0.7
ΔDD	−232.0 ± 793.9	0.38
ΔHCO_3_	−0.8 ± 3.2	0.5
ΔPaO_2_	21.7 ± 24.5	0.03
ΔPaCO_2_	−1.9 ± 4.2	0.29
ΔFiO_2_	−21.0 8 ± 19.6	0.01
ΔP/F	222.8 ± 86.0	<0.001

CRP: reactive C protein; DD: d-dimer: IL-6: interleukin-6; LDH: lactate dehydrogenase; LYM: lymphocytes count; WBC: white blood cells count; HCO_3_: bicarbonate; PaO_2_: partial pressure of oxygen; PaCO_2_: partial pressure of carbon dioxide; FiO_2_: fraction of inspired oxygen; P/F: PaO_2_/FiO_2_ ratio.

## Data Availability

The data presented in this study are available on request from the corresponding author. The data are not publicly available due to the privacy policy of the centers involved in the study.

## References

[1] Wang D., Hu B., Hu C., Zhu F., Liu X., Zhang J., Wang B., Xiang H., Cheng Z., Xiong Y. (2020). Clinical Characteristics of 138 Hospitalized Patients With 2019 Novel Coronavirus-Infected Pneumonia in Wuhan, China. JAMA.

[2] Xu X.W., Wu X.X., Jiang X.G., Xu K.J., Ying L.J., Ma C.L., Li S.B., Wang H.Y., Zhang S., Gao H.N. (2020). Clinical findings in a group of patients infected with the 2019 novel coronavirus (SARS-Cov-2) outside of Wuhan, China: Retrospective case series. BMJ.

[3] Zhu N., Zhang D., Wang W., Li X., Yang B., Song J., Zhao X., Huang B., Shi W., Lu R. (2020). China Novel Coronavirus I, Research T. A Novel Coronavirus from Patients with Pneumonia in China, 2019. N. Engl. J. Med..

[4] Lu H., Stratton C.W., Tang Y.W. (2020). Outbreak of pneumonia of unknown etiology in Wuhan, China: The mystery and the miracle. J. Med. Virol..

[5] Chen N., Zhou M., Dong X., Qu J., Gong F., Han Y., Qiu Y., Wang J., Liu Y., Wei Y. (2020). Epidemiological and clinical characteristics of 99 cases of 2019 novel coronavirus pneumonia in Wuhan, China: A descriptive study. Lancet.

[6] Huang C., Wang Y., Li X., Ren L., Zhao J., Hu Y., Zhang L., Fan G., Xu J., Gu X. (2020). Clinical features of patients infected with 2019 novel coronavirus in Wuhan, China. Lancet.

[7] Batah S.S., Fabro A.T. (2021). Pulmonary pathology of ARDS in COVID-19: A pathological review for clinicians. Respir. Med..

[8] Doglioni C., Ravaglia C., Chilosi M., Rossi G., Dubini A., Pedica F., Piciucchi S., Vizzuso A., Stella F., Maitan S. (2021). Covid-19 Interstitial Pneumonia: Histological and Immunohistochemical Features on Cryobiopsies. Respiration.

[9] Hanley B., Lucas S.B., Youd E., Swift B., Osborn M. (2020). Autopsy in suspected COVID-19 cases. J. Clin. Pathol..

[10] Xu Z., Shi L., Wang Y., Zhang J., Huang L., Zhang C., Liu S., Zhao P., Liu H., Zhu L. (2020). Pathological findings of COVID-19 associated with acute respiratory distress syndrome. Lancet Respir. Med..

[11] Habashi N.M., Camporota L., Gatto L.A., Nieman G. (2021). Functional pathophysiology of SARS-CoV-2-induced acute lung injury and clinical implications. J. Appl. Physiol..

[12] Bradley B.T., Maioli H., Johnston R., Chaudhry I., Fink S.L., Xu H., Najafian B., Deutsch G., Lacy J.M., Williams T. (2020). Histopathology and ultrastructural findings of fatal COVID-19 infections in Washington State: A case series. Lancet.

[13] Rubin G.D., Ryerson C.J., Haramati L.B., Sverzellati N., Kanne J.P., Raoof S., Schluger N.W., Volpi A., Yim J.J., Martin I.B.K. (2020). The Role of Chest Imaging in Patient Management During the COVID-19 Pandemic: A Multinational Consensus Statement From the Fleischner Society. Chest.

[14] Roshkovan L., Chatterjee N., Galperin-Aizenberg M., Gupta N., Shah R., Barbosa E.M., Simpson S., Cook T., Nachiappan A., Knollmann F. (2020). The Role of Imaging in the Management of Suspected or Known COVID-19 Pneumonia. A Multidisciplinary Perspective. Ann. Am. Thorac. Soc..

[15] Cartocci G., Colaiacomo M.C., Lanciotti S., Andreoli C., De Cicco M.L., Brachetti G., Pugliese S., Capoccia L., Tortora A., Scala A. (2021). Correction to: Chest CT for early detection and management of coronavirus disease (COVID-19): A report of 314 patients admitted to Emergency Department with suspected pneumonia. Radiol. Med..

[16] Islam N., Ebrahimzadeh S., Salameh J.P., Kazi S., Fabiano N., Treanor L., Absi M., Hallgrimson Z., Leeflang M.M., Hooft L. (2021). Cochrane C-DTAG. Thoracic imaging tests for the diagnosis of COVID-19. Cochrane Database Syst. Rev..

[17] Peng Q.Y., Wang X.T., Zhang L.N. (2020). Chinese Critical Care Ultrasound Study G. Findings of lung ultrasonography of novel corona virus pneumonia during the 2019-2020 epidemic. Intensive Care Med..

[18] Soldati G., Smargiassi A., Inchingolo R., Buonsenso D., Perrone T., Briganti D.F., Perlini S., Torri E., Mariani A., Mossolani E.E. (2020). Proposal for International Standardization of the Use of Lung Ultrasound for Patients With COVID-19: A Simple, Quantitative, Reproducible Method. J. Ultrasound Med..

[19] Tung-Chen Y., Marti de Gracia M., Diez-Tascon A., Alonso-Gonzalez R., Agudo-Fernandez S., Parra-Gordo M.L., Ossaba-Velez S., Rodriguez-Fuertes P., Llamas-Fuentes R. (2020). Correlation between Chest Computed Tomography and Lung Ultrasonography in Patients with Coronavirus Disease 2019 (COVID-19). Ultrasound Med. Biol..

[20] Bhoi S., Sahu A.K., Mathew R., Sinha T.P. (2021). Point-of-care ultrasound in COVID-19 pandemic. Postgrad Med. J..

[21] Mei F., Bonifazi M., Menzo S., Di Marco Berardino A., Sediari M., Paolini L., Re A., Gonnelli F., Duranti C., Grilli M. (2020). First Detection of SARS-CoV-2 by Real-Time Reverse Transcriptase-Polymerase Chain Reaction Assay in Pleural Fluid. Chest.

[22] Falster C., Jacobsen N., Wulff Madsen L., Dahlerup Rasmussen L., Davidsen J.R., Christie Knudtzen F., Nielsen S.L., Johansen I.S., Laursen C.B. (2021). Lung ultrasound may be a valuable aid in decision making for patients admitted with COVID-19 disease. Eur. Clin. Respir. J..

[23] Amir O., Rappaport D., Zafrir B., Abraham W.T. (2013). A novel approach to monitoring pulmonary congestion in heart failure: Initial animal and clinical experiences using remote dielectric sensing technology. Congest Heart Fail..

[24] Amir O., Ben-Gal T., Weinstein J.M., Schliamser J., Burkhoff D., Abbo A., Abraham W.T. (2017). Evaluation of remote dielectric sensing (ReDS) technology-guided therapy for decreasing heart failure re-hospitalizations. Int. J. Cardiol..

[25] Amir O., Azzam Z.S., Gaspar T., Faranesh-Abboud S., Andria N., Burkhoff D., Abbo A., Abraham W.T. (2016). Validation of remote dielectric sensing (ReDS) technology for quantification of lung fluid status: Comparison to high resolution chest computed tomography in patients with and without acute heart failure. Int. J. Cardiol..

[26] Bensimhon D., Alali S.A., Curran L., Gelbart E., Garman D.W.V., Taylor R., Chase P., Peacock W.F. (2021). The use of the reds noninvasive lung fluid monitoring system to assess readiness for discharge in patients hospitalized with acute heart failure: A pilot study. Heart Lung.

[27] Uriel N., Sayer G., Imamura T., Rodgers D., Kim G., Raikhelkar J., Sarswat N., Kalantari S., Chung B., Nguyen A. (2018). Relationship Between Noninvasive Assessment of Lung Fluid Volume and Invasively Measured Cardiac Hemodynamics. J. Am. Heart Assoc..

[28] Groarke J.D., Stevens S.R., Mentz R.J., Cooper L.B., Vader J.M., AbouEzzeddine O.F., Grodin J.L., Joyce E., Anstrom K.J., Felker G.M. (2018). Clinical Significance of Early Fluid and Weight Change During Acute Heart Failure Hospitalization. J. Card Fail..

[29] Li K., Fang Y., Li W., Pan C., Qin P., Zhong Y., Liu X., Huang M., Liao Y., Li S. (2020). CT image visual quantitative evaluation and clinical classification of coronavirus disease (COVID-19). Eur. Radiol..

[30] Lala A., Barghash M.H., Giustino G., Alvarez-Garcia J., Konje S., Parikh A., Ullman J., Keith B., Donehey J., Mitter S.S. (2020). Early use of remote dielectric sensing after hospitalization to reduce heart failure readmissions. ESC Heart Fail..

[31] Gambardella C., Pagliuca R., Pomilla G., Gambardella A. (2020). COVID-19 risk contagion: Organization and procedures in a South Italy geriatric oncology ward. J. Geriatr. Oncol..

[32] Tolone S., Gambardella C., Brusciano L., Del Genio G., Lucido F.S., Docimo L. (2020). Telephonic triage before surgical ward admission and telemedicine during COVID-19 outbreak in Italy. Effective and easy procedures to reduce in-hospital positivity. Int. J. Surg..

